# SMYD5-BRD4 Interaction Drives Hepatocellular Carcinoma Progression: A Combined in Silico and Experimental Analysis

**DOI:** 10.3390/ph18081105

**Published:** 2025-07-25

**Authors:** Mingye Hu, Shiji Chen, Yumiao Zhen, Xin Wang, Yiwen Zhong, Xiaoxu Liang, Cheong-Meng Chong, Hai-Jing Zhong

**Affiliations:** 1 State Key Laboratory of Bioactive Molecules and Druggability Assessment, International Cooperative Laboratory of Traditional Chinese Medicine Modernization and Innovative Drug Development of Chinese Ministry of Education (MOE) of China, School of Pharmacy, Jinan University, Guangzhou 510632, China; hmyyye712@163.com (M.H.); chen94599@163.com (S.C.); zymiu4@163.com (Y.Z.); wangxin3201194992@163.com (X.W.); zhongyiwen98@163.com (Y.Z.); 2 School of Arts and Sciences, Guangzhou Maritime University, Guangzhou 510725, China; 3 State Key Laboratory of Mechanism and Quality of Chinese Medicine, Institute of Chinese Medical Sciences, University of Macau, Macao SAR, China

**Keywords:** LIHC, SMYD5, BRD4, protein–protein interaction, epigenetic

## Abstract

**Background/Objectives:** Hepatocellular carcinoma (LIHC) poses significant challenges due to limited targeted therapeutic options. This study investigates SMYD5, an oncogene implicated in the pathogenesis of LIHC, and its interaction with the BRD4 protein. **Methods**: We employed bioinformatics analyses alongside experimental validations to assess SMYD5 expression across various cancers, particularly LIHC. This included survival analysis, protein expression studies, and functional assays to understand the role of SMYD5 in LIHC progression. **Results**: Our findings demonstrate that SMYD5 expression is markedly elevated in LIHC tumor tissues compared to normal liver tissues. Moreover, high levels of SMYD5 correlate with poor overall survival and disease-free survival rates in LIHC patients. Functional assays indicate that the knockdown of SMYD5 significantly inhibits cell proliferation and increases apoptosis in LIHC cell lines. Additionally, a notable interaction between SMYD5 and BRD4 was identified, suggesting a potential therapeutic target in the SMYD5-BRD4 axis. **Conclusions**: These findings collectively establish SMYD5 as a molecular driver in LIHC pathology and identify the SMYD5-BRD4 interaction axis as a promising therapeutic target for future drug development.

## 1. Introduction

Liver hepatocellular carcinoma (LIHC) constitutes a significant global health burden [1]. The rapid progression and heterogeneity of LIHC pose significant challenges for clinical treatment. Although targeted therapies such as sorafenib and lenvatinib are available, their effectiveness is often limited for many patients, typically resulting in only a few additional months of survival [2]. Furthermore, while immunotherapy has shown promise for certain LIHC patients, not all individuals respond favorably to immune checkpoint inhibitors [3]. As a result, the treatment of LIHC continues to face significant hurdles, including treatment resistance, limitations in immunotherapy, and considerable individual differences among patients [4].

SET and MYND domain-containing protein family member 5 (SMYD5) is one of five members of the SMYD protein family (SMYD1–5). SMYD5 functions as a lysine methyltransferase that predominantly catalyzes the trimethylation of lysine 22 (K22me3) on ribosomal protein L40 (RPL40/eL40), thereby regulating translational output and the associated biological processes in LIHC [5]. Recent studies have primarily focused on the role of SMYD5 as a ribosomal methyltransferase and its significance in cancer [6,7]. Notably, SMYD5 has been implicated in the malignant progression of gastric cancer and LIHC by promoting the trimethylation of rpL40K22, which enhances mRNA translation output [8]. Disruption of histone lysine methylation dynamics and the resulting transcriptional dysregulation are established contributors to tumorigenesis. Furthermore, inhibition of either SMYD5 or its substrate rpL40K22me3 has been shown to significantly reduce the metastasis of gastric cancer cells and increase sensitivity to PI3K-mTOR inhibitors in gastric cancer [8]. BRD4 is a member of the bromodomain and extra-terminal (BET) family that functions as a critical transcriptional coactivator, primarily by recognizing acetylated lysine residues on histones and recruiting P-TEFb to promote transcriptional elongation. BRD4-mediated transcriptional regulation is essential for the expression of oncogenes and has been implicated in various malignancies, including LICH. In parallel, SMYD5 is a well-characterized histone methyltransferase, known since its discovery to mediate methylation at the histone H3K36, contributing to chromatin remodeling and gene expression regulation [9].

However, systematic pan-cancer analyses of SMYD5, particularly regarding its molecular mechanisms and epigenetic roles in tumorigenesis and progression, are still lacking. In this study, we examined SMYD5 across multiple cancers, with a focus on LIHC, and investigated its interaction with BRD4 in LIHC progression. By combining bioinformatics analyses with experimental validation, our work aims to provide novel insights and potential therapeutic targets for the prognostic evaluation and targeted treatment of LIHC.

## 2. Results

### 2.1. Analysis of SMYD5 Expression Across Cancer Types

**Figure 1:**
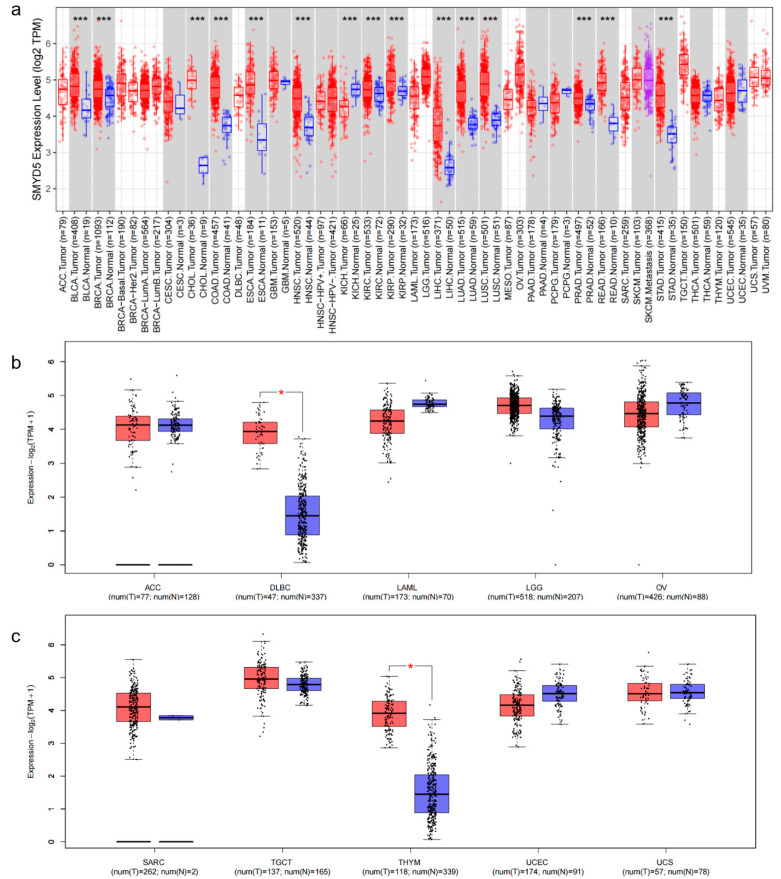
Assessment of SMYD5 expression differences between tumors and normal tissues across various cancer types. (**a**) Pan-cancer analysis of SMYD5 expression in tumors (red) vs. normal tissues (blue) using the TIMER 2.0 tool. (**b**) The expression levels of the SMYD5 gene were assessed in specific cancer types (red), including ACC, DLBC, LAML, LGG, and OV vs. normal tissues (purple) using the GEPIA2 tool. (**c**) The expression levels of the SMYD5 gene were assessed in specific cancer types, including SARC, TGCT, THYM, UCEC, and UCS using the GEPIA2 tool. Statistical significance is calculated through one-way ANOVA and indicated by * *p* < 0.05, and *** *p* < 0.001.

To explore the involvement of SMYD5 in cancers, we used the TIMER2.0 platform to assess its expression across 33 cancer types from the TCGA dataset (Figure 1a). However, SMYD5 expression data for normal tissues were not available for several cancers in TCGA, such as adrenocortical carcinoma (ACC), diffuse large B-cell lymphoma (DLBC), acute myeloid leukemia (LAML), lower-grade glioma (LGG), ovarian cancer (OV), sarcoma (SARC), testicular germ cell tumors (TGCT), thymoma (THYM), uterine corpus endometrial carcinoma (UCEC), and uterine carcinosarcoma (UCS). To overcome this gap, we utilized the GEPIA2 tool to complement and confirm the SMYD5 expression findings (Figure 1b,c) [10,11,12,13]. Our analysis demonstrated that SMYD5 expression was notably higher in tumor tissues compared to normal counterparts in several cancer types, including bladder cancer (BLCA), breast cancer (BRCA), cholangiocarcinoma (CHOL), colon adenocarcinoma (COAD), diffuse large B-cell lymphoma (DLBC), esophageal carcinoma (ESCA), head and neck squamous cell carcinoma (HNSC), kidney renal clear cell carcinoma (KIRC), kidney renal papillary cell carcinoma (KIRP), liver hepatocellular carcinoma (LIHC), lung adenocarcinoma (LUAD), lung squamous cell carcinoma (LUSC), prostate adenocarcinoma (PRAD), rectal adenocarcinoma (READ), stomach adenocarcinoma (STAD), and thymoma (THYM). Conversely, patients with kidney chromophobe carcinoma (KICH) exhibited lower SMYD5 expression levels relative to normal individuals. Furthermore, paired clinical samples demonstrated SMYD5 gene expression was significantly increased in multiple tumor types relative to their matched normal tissues (Supplementary Figure S1).

### 2.2. Elevated SMYD5 Levels Correlate with Poor Prognosis in LIHC Patients

**Figure 2:**
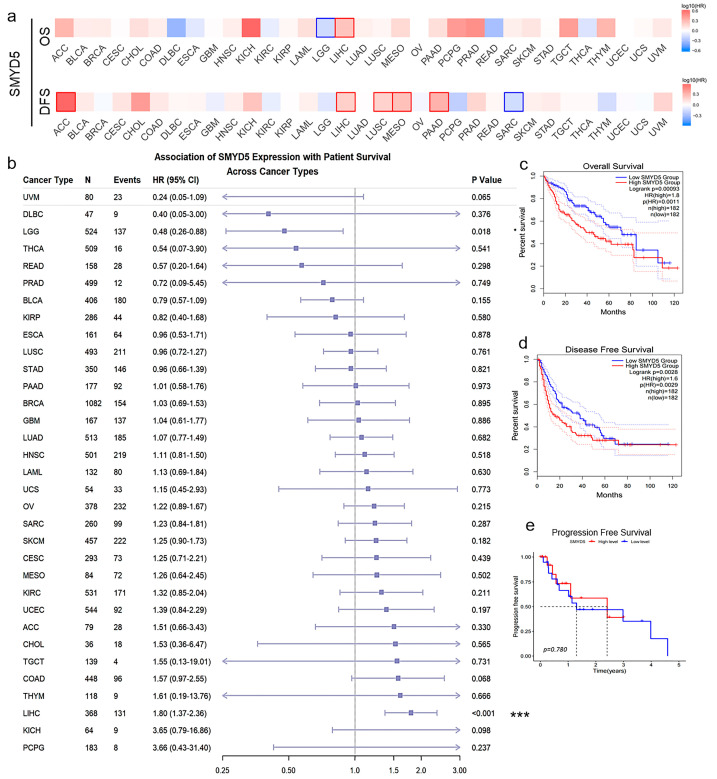
Prognostic analysis based on SMYD5 expression levels. (**a**) OS and DFS analysis of SMYD5 expression using data from the TCGA dataset. (**b**) The association between SMYD5 expression and survival outcomes across different cancer types was assessed using Cox regression analysis. (**c**) Kaplan–Meier OS analysis of SMYD5 in LIHC (*p* = 0.0011). (**d**) DFS analysis of SMYD5 in LIHC (*p* = 0.0029). (**e**) PFS analysis of SMYD5 in LIHC (*p* = 0.780). Statistical significance is indicated by * *p* < 0.05 and *** *p* < 0.001.

Pan-cancer analyses revealed a significant positive correlation between elevated SMYD5 expression and poor overall survival (OS) and disease-free survival (DFS) across multiple cancer types, with the strongest association observed in LIHC (Figure 2a). A Cox regression analysis further identified that an elevated SMYD5 expression had the strongest correlation with poor survival outcomes in LIHC (*p* < 0.001, Figure 2b). A Kaplan–Meier analysis of clinical data demonstrated that high SMYD5 expression was consistently associated with poor OS, DFS, and progression-free survival (PFS) in LIHC (Figure 2c–e), although the prognostic value of PFS was relatively limited.

Comprehensive clinicopathological and survival analyses were conducted to evaluate the prognostic significance of SMYD5 expression in LIHC patients. Analysis of survival rates in LIHC patients based on SMYD5 expression and clinical factors showed one-year, three-year, and five-year survival rates of 87.1%, 75.8%, and 67.5%, respectively (Supplementary Figure S2a,b). Receiver operating characteristic (ROC) curves (Supplementary Figure S2c) indicated reasonable predictive accuracy for short-term survival, while long-term prognostic performance was limited. Univariate and multivariate analyses (Supplementary Figure S2d,e) identified the clinical stage as a key prognostic factor, with SMYD5 expression also making a significant contribution to patient outcomes. Notably, SMYD5 expression differed significantly between high and low expression groups across pathological grades (G grade) and T stages in LIHC patients (Supplementary Figure S2f–i), with *p* values of 0.0011 for G1 vs. G3, 0.00055 for G1 vs. G4, 0.0037 for G2 vs. G3, and 0.0021 for G2 vs. G4. For clinical stages, the *p* values were 0.033 for Stage I vs. Stage II, 0.026 for Stage I vs. Stage III, and 0.01 for T1 vs. T2. These results suggest that SMYD5 may serve as a valuable clinical indicator for LIHC staging.

### 2.3. Role of SMYD5 in Tumor Immune Microenvironment in LIHC

To highlight the potential role of SMYD5 in shaping the tumor immune microenvironment, we analyzed the correlation between SMYD5 expression and tumor-infiltrating lymphocytes (TILs). The results presented in the analysis highlight the significant correlation between SMYD5 expression and TILs, as illustrated in Supplementary Figure S3a. The scatter plot reveals a positive relationship (R = 0.17, *p* = 0.0024), indicating that higher SMYD5 levels are associated with an increased TIL presence, suggesting a potential role for SMYD5 in modulating the tumor immune microenvironment. This is further supported by the violin plot in Supplementary Figure S3b, which demonstrates that SMYD5 expression significantly influences TIL scores, with the highest TIL levels found in samples exhibiting elevated SMYD5 expression (*p* < 0.001). In Supplementary Figure S3c, partial correlation analyses indicate that SMYD5 expression correlates positively with various immune cell types, such as B cells and CD8+ T cells, adding depth to the findings by suggesting that SMYD5 might facilitate immune infiltration and activity within tumors. Supplementary Figure S3d presents a heatmap illustrating the correlation coefficients among different immune-related markers, with SMYD5 showing notable positive correlations with other immune modulators, such as CD276 and TNFSF9. This comprehensive analysis suggests that targeting SMYD5 may enhance immune responses against tumors, highlighting its potential as a therapeutic target in cancer immunotherapy. Overall, these findings underscore the importance of SMYD5 in shaping the tumor immune landscape and its implications in improving anti-tumor immune responses.

### 2.4. Characterization of SMYD5-Related Pathways in LIHC

To understand the role of SMYD5 in regulating LIHC molecular pathways, Gene Ontology (GO), Kyoto Encyclopedia of Genes and Genomes (KEGG), and Gene Set Enrichment Analysis (GSEA) were employed (Supplementary Figure S4). The GO analysis revealed that SMYD5 is predominantly involved in the regulation of biological processes and molecular functions, particularly embryonic skeletal development and ion channel activities associated with neuromodulation. The KEGG enrichment analysis indicated that SMYD5 is mainly implicated in the neuroactive ligand–receptor interaction pathway. Furthermore, GSEA demonstrated that lower SMYD5 expression is associated with the downregulation of pathways related to fatty acid metabolism and primary bile acid biosynthesis. To further elucidate the potential regulatory network of SMYD5 in LIHC, comprehensive co-expression and functional analyses were performed. We identified ten co-expressed genes associated with SMYD5 in this study and discovered 100 differentially expressed genes correlated with SMYD5 in LIHC (Supplementary Figure S5). These genes were then visualized on a heatmap, which highlights their expression patterns. A co-expression analysis revealed that the expression level of the growth hormone receptor (GHR) was negatively correlated with SMYD5 in LIHC, and a KEGG analysis indicated that SMYD5 was associated with the secretion of GnRH (Supplementary Figure S4b). GHR belongs to a superfamily of transmembrane proteins, including the prolactin receptor and some cytokine receptors, and it is abundantly expressed in the liver [14,15].

### 2.5. SMYD5 Knockdown Inhibits Proliferation and Promotes Apoptosis in LIHC Cells

**Figure 3:**
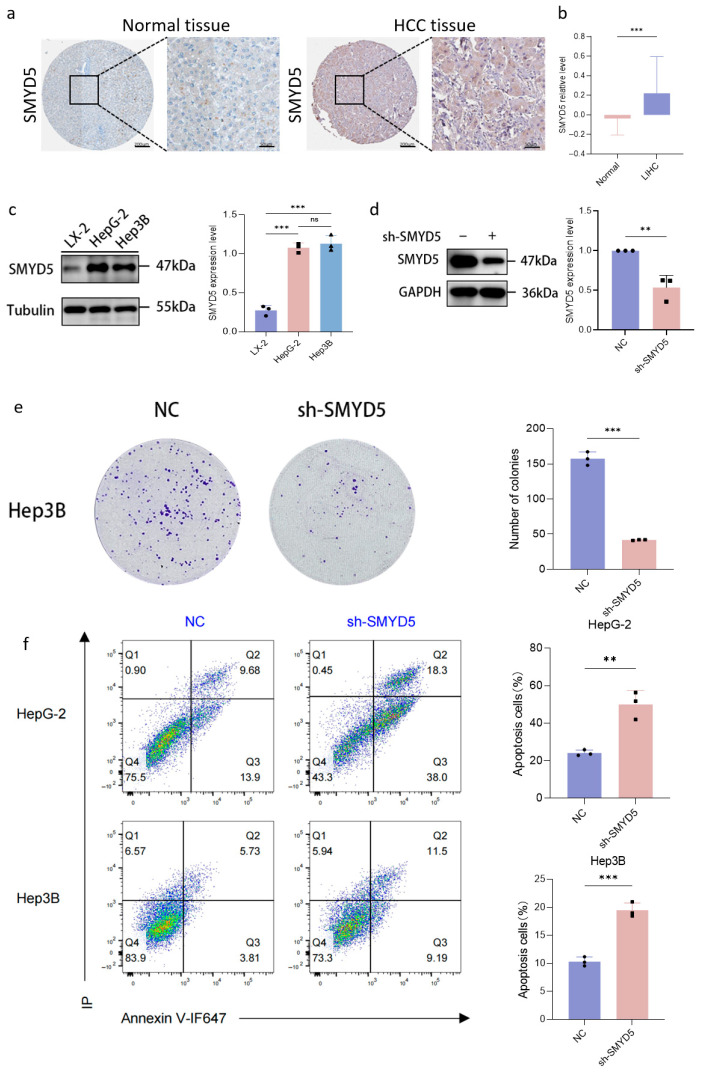
SMYD5 expression is associated with adverse prognosis and drives LIHC progression through BRD4 regulation. (**a**) SMYD5 expression in normal and tumor tissues from HPA dataset. The boxed region in the image is magnified for detail. (**b**) CPTAC data showing an increased SMYD5 protein level in LIHC tissues. (**c**) SMYD5 expression across hepatic cell lines. Each point on the bar chart represents an individual sample. “ns” indicates no significant difference. (**d**) Western blot and densitometric analysis demonstrated that SMYD5 protein expression was significantly decreased in sh-SMYD5-transfected Hep3B cells compared to the control group (sh-NC) (*p* < 0.01), indicating effective knockdown. Each point on the bar chart represents an individual sample. (**e**) Colony formation in Hep3B cells. Each point on the bar chart represents an individual sample. (**f**) Knockdown of SMYD5 expression in HepG-2 and Hep3B cell lines promotes cell apoptosis in both early and late phases. Each point on the bar chart represents an individual sample. Data are represented as mean ± SD. Statistical significance is indicated by ** *p* < 0.01, and *** *p* < 0.001.

To confirm the overexpression of SMYD5 in LIHC, we analyzed immunohistochemical staining and protein expression of LIHC tumor tissues and normal tissues from the Human Protein Atlas (HPA) and Clinical Proteomic Tumor Analysis Consortium (CPTAC). The results showed a marked increase in SMYD5 protein levels in LIHC tumor tissues compared to normal tissues (Figure 3a,b). We further assessed the SMYD5 expression in LIHC cell lines (HepG2 and Hep3B) and in the normal hepatic stellate cell line LX-2. Consistently, SMYD5 expression was significantly higher in HepG2 and Hep3B cells than in LX-2 cells (Figure 3c). To further investigate the functional role of SMYD5 in LIHC progression, we generated Hep3B cells with a stable SMYD5 knockdown using lentivirus-mediated short hairpin RNA (shRNA). As shown in Figure 3d, SMYD5 protein expression was significantly lower in the sh-SMYD5 group than in the negative control (sh-NC) group according to Western blot results. Additionally, SMYD5 depletion significantly inhibited the proliferation of Hep3B cells, as evidenced by the reduced colony formation (Figure 3e, *p* < 0.01).

To further investigate the mechanism underlying SMYD5-mediated oncogenic effects, we performed a flow cytometric analysis using Annexin V-IF647/PI double staining in both HepG2 and Hep3B cell lines. The results demonstrated that SMYD5 knockdown significantly increased the proportion of apoptotic cells in both early and late apoptotic phases compared to control groups (Figure 3f). Specifically, the total apoptotic rate increased from 24.1 ± 0.6% to 50.0 ± 7.4% in HepG2 cells and from 10.3 ± 0.8% to 19.5 ± 1.3% in Hep3B cells following SMYD5 silencing (*p* < 0.001), suggesting that SMYD5 may promote LIHC progression partly through the inhibition of programmed cell death pathways.

### 2.6. Interaction Between SMYD5 and BRD4 in LIHC

**Figure 4:**
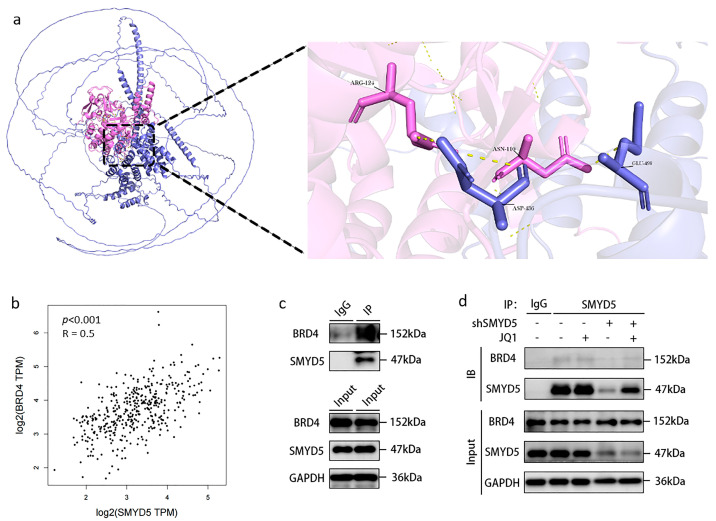
Structural insights, molecular associations, and functional effects of SMYD5 and BRD4 in LIHC. (**a**) Positive correlation between SMYD5 and BRD4 expression levels in LIHC tumor and normal tissues. (**b**) Structure of the SMYD5-BRD4 complex predicted by AlphaFold3. (**c**) Co-IP assay indicated SMYD5 existed in combination with BRD4 in vitro. (**d**) Co-IP assay demonstrated the combined blocking effect of (+)-JQ1 and shSMYD5 on SMYD5-BRD4 binding.

To elucidate potential interaction partners and regulatory mechanisms of SMYD5 in LIHC, we conducted a comprehensive protein–protein interaction (PPI) analysis. This analysis identified 50 proteins closely associated with SMYD5 (Supplementary Figure S6). Within the PPI network, BRD4, TP53, UBA52, RBBP4, KDM6A, and KDM5C, etc., occupy central positions. Notably, BRD4 is highlighted due to its key role as an acetyltransferase in histone epigenetic modification, a primary focus of our group’s cancer drug development efforts [16]. As a chromatin reader, BRD4 recognizes and binds to acetylated histones, thereby facilitating the transmission of epigenetic memory during cell division and regulating transcription [17,18,19,20,21]. Given the roles of both BRD4 and SMYD5 in histone modification, we hypothesized a functional interaction between these two proteins. To investigate this hypothesis, we employed AlphaFold3 for structure prediction and utilized PyMOL (3.1.3) for molecular visualization. We selected one of the highest confidence models from AlphaFold3 (model-0: ipTM = 0.29, pTM = 0.34) to visualize the BRD4–SMYD5 interaction interface (BRD4 in violet, SMYD5 in pink; Figure 4a). Furthermore, analysis using the GEPIA2 tool revealed a positive correlation between SMYD5 and BRD4 expression levels in LIHC (Figure 4b). To validate the interaction between SMYD5 and BRD4, we conducted co-immunoprecipitation (co-IP) experiments in Hep3B cells, which demonstrated a direct interaction between SMYD5 and BRD4 (Figure 4c). The compound (+)-JQ1, known as a potent and selective inhibitor of BRD4, interferes with BET protein binding to acetylated lysines on histones and transcription factors, thus suppressing tumor growth. Interestingly, our results indicate that SMYD5 knockdown further enhances this inhibitory effect of (+)-JQ1 (Figure 4d), suggesting that this compound can effectively disrupt the SMYD5–BRD4 interaction.

In addition to BRD4, we also investigated the interactions involving TP53. Given the frequent mutations of TP53 in LIHC, we speculated that alterations in TP53 may significantly affect its interactions with SMYD5 and contribute to downstream regulatory pathways. Our findings from the IP analysis confirm a direct interaction between SMYD5 and p53 (Figure S7). This interaction implies that SMYD5 could play a crucial role in modulating p53 functionality, potentially influencing essential cellular processes, including cell cycle progression and apoptosis.

### 2.7. Targeting SMYD5 Enhances BRD4 Inhibition Efficacy in Suppressing LIHC Migration and Promoting Apoptosis

**Figure 5:**
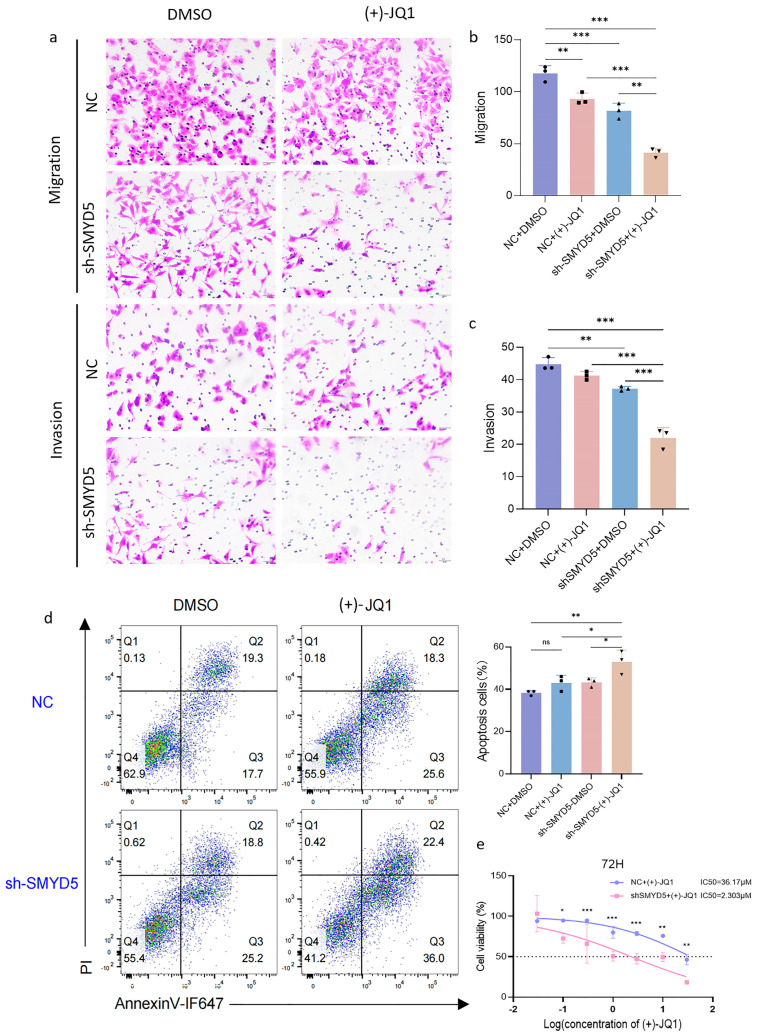
Impact of SMYD5 inhibition on migration, invasion, and apoptosis of Hep3B cells treated with BRD4 inhibitor (+)-JQ1. (**a**) Representative images of migration and invasion assays in Hep3B cells treated with DMSO or (+)-JQ1, comparing negative control (NC) and SMYD5-silenced (sh-SMYD5) groups. (**b**) Quantification of migratory capabilities, indicating a significant reduction in migration rates with SMYD5 silencing and a further decrease upon JQ1 treatment. (**c**) Invasion assay quantification showing reduced invasion in sh-SMYD5 cells treated with (+)-JQ1 compared to controls. (**d**) Flow cytometric analysis assessing apoptosis in Hep3B cells after treatment with DMSO or (+)-JQ1. The percentages of apoptotic cells are shown in the quadrants, highlighting increased apoptosis in the sh-SMYD5 group upon JQ1 treatment. (**e**) Cell viability analysis over 72 h demonstrating the enhanced sensitivity of sh-SMYD5 cells to (+)-JQ1, with lower IC_50_ compared to NC cells. Data are represented as mean ± SD. Statistical significance is indicated as * *p* < 0.05, ** *p* < 0.01, and *** *p* < 0.001.

To elucidate the role of SMYD5 in LIHC aggressiveness and assess the therapeutic potential of its inhibition, particularly in combination with a BRD4 blockade, migration and invasion assays were conducted. The results showed that SMYD5 silencing significantly reduced the migratory and invasive capacities of Hep3B cells, with migration and invasion rates decreasing by 30.68 ± 6.03% and 14.87 ± 1.45%, respectively (Figure 5a–c). Notably, the addition of (+)-JQ1 to SMYD5-silenced Hep3B cells resulted in a further significant reduction in both migration and invasion abilities. Moreover, the treatment of SMYD5 silencing Hep3B cells with (+)-JQ1 for 24 h led to a pronounced increase in the proportion of apoptotic cells, as determined by a flow cytometry analysis (Figure 5d). Importantly, SMYD5 inhibition enhanced the sensitivity of Hep3B cells to (+)-JQ1, resulting in a significant inhibition on proliferation compared to (+)-JQ1 treatment alone (Figure 5e). Collectively, these results demonstrate that the combined inhibition of SMYD5 and BRD4 not only synergistically suppresses migration and invasion, but also promotes apoptosis in Hep3B cells, suggesting that targeting SMYD5 may enhance the anti-tumor efficacy of BRD4 inhibition and reduce the metastatic potential of LIHC cells.

### 2.8. Impact of SMYD5 and BRD4 Targeting on Cell Cycle Regulators and Methylation Modification in Lihc Progression

**Figure 6:**
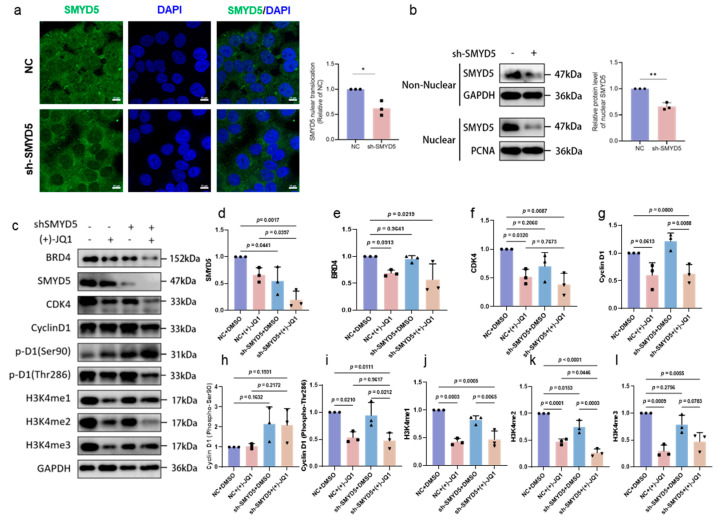
Effects of SMYD5 knockdown and (+)-JQ1 treatment on cell cycle regulators and methylation modification in LIHC cells. (**a**) Representative immunofluorescence images showing SMYD5 localization in Hep3B cells, with DAPI staining for nuclei (scale bar: 10 μm). Quantification of SMYD5 nuclear translocation relative to control (NC); results indicate a significant decrease in nuclear localization upon sh-SMYD5. (**b**) Western blot analysis and quantification demonstrate that the relative level of SMYD5 in the nucleus is significantly decreased following knockdown. (**c**) Western blot analysis illustrates changes in protein expression levels for BRD4, SMYD5, CDK4, cyclin D1, and histone methylation marks (H3K4me1, H3K4me2, and H3K4me3) among different treatment groups: NC, sh-SMYD5, and (+)-JQ1. Relative quantification of protein levels is shown in (**d**–**l**), highlighting significant variations between treatments. Data are represented as mean ± SD. Statistical significance is indicated * *p* < 0.05, ** *p* < 0.01.

Our study further elucidates the localization and regulatory roles of SMYD5 in Hep3B cells. We found that SMYD5 is distributed in both the cytoplasm and nucleus (Figure 6a). Notably, the knockdown of SMYD5 significantly reduced its nuclear localization (Figure 6a,b), suggesting that SMYD5 may play an important role in nuclear functions associated with tumorigenesis. Western blot analysis revealed critical changes in the expression levels of several key proteins involved in cell cycle regulation, particularly focusing on SMYD5 and BRD4. The data demonstrated a significant reduction in SMYD5 levels upon knockdown, indicating its pivotal role in cellular functions associated with tumorigenesis (*p* = 0.0017, *p* = 0.0397). Additionally, treatment with the BRD4 inhibitor (+)-JQ1 resulted in a marked decrease in BRD4 expression, especially in the context of SMYD5 suppression (*p* = 0.0219) (Figure 6c–e). This suggests a synergistic relationship between SMYD5 and BRD4 that enhances our understanding of their interplay in oncogenic pathways. We also observed a significant decrease in the CDK4 expression when both shSMYD5 and (+)-JQ1 were used (*p* = 0.0087), highlighting the downstream effects of targeting both proteins (Figure 6f). While Cyclin D1 expression showed a downward trend, it did not reach statistical significance (*p* = 0.0800). However, the phosphorylated form of Cyclin D1 at Thr286 significantly decreased with combined treatment (*p* = 0.0210), indicating specific regulatory mechanisms by which SMYD5 and BRD4 modulate cell cycle progression (Figure 6g–i). The analysis of histone methylation marks (Figure 6j–l) reveals significant changes, with reductions in H3K4me1, H3K4me2, and H3K4me3 observed upon dual treatment. These findings indicate that targeting SMYD5 and BRD4 not only affects protein levels but also modifies the methylation landscape, which is crucial for regulating gene expression in LIHC.

## 3. Discussion

The complexity of clinical pathogenetic factors complicates the treatment of LIHC, underscoring the need for innovative therapeutic targets [22,23]. Our study identifies SMYD5, a protein-lysine N-trimethyltransferase, as a critical player in LIHC progression. We established that SMYD5 expression is significantly elevated in tumor tissues compared to corresponding normal tissues, confirming its potential role in oncogenesis (Figure 1). Furthermore, our pan-cancer analyses demonstrated a robust positive correlation between heightened SMYD5 levels and poor OS and DFS, especially among LIHC patients (Figure 2). These associations suggest that SMYD5 not only affects tumor biology but also impacts the tumor microenvironment and patient outcomes, aligning with earlier findings [5,24]. Elevated SMYD5 levels are linked to adverse clinical outcomes due to their role in modifying key histone marks that influence gene expression. Specifically, SMYD5 catalyzes the trimethylation of histone H3 at lysine 36, which is associated with transcriptional activation in cancer [25]. Moreover, members of the SMYD family have been shown to affect DNA repair pathways through histone modification, potentially increasing the tumor mutation burden [26]. This correlation indicates that elevated SMYD5 expression induces epigenetic alterations that disrupt cellular homeostasis, promoting tumorigenesis.

Our analysis confirmed that SMYD5 is overexpressed in LIHC tissues compared to adjacent normal tissues, as established through immunohistochemical staining and protein expression profiling using data from the Human Protein Atlas (HPA) and the Clinical Proteomic Tumor Analysis Consortium (CPTAC) (Figure 3a,b). This consistent upregulation of SMYD5 in both LIHC tumor tissues and cell lines (HepG2 and Hep3B) relative to the normal hepatic stellate cell line LX-2 (Figure 3c) underscores its potential role as a promoter of hepatocarcinogenesis.

To assess the functional role of SMYD5 in LIHC progression, we applied shRNA to achieve the stable knockdown of SMYD5 in Hep3B cells. Quantitative results from the Western blot analysis demonstrated a significant reduction in SMYD5 protein levels in the sh-SMYD5 group compared to the negative control (sh-NC) group (Figure 3d). Importantly, this knockdown resulted in a marked inhibition of cell proliferation, as evidenced by diminished colony formation capabilities (Figure 3e). These results are consistent with prior findings that indicate a functional dependency of cancer cells on elevated SMYD5 levels for growth and survival.

Furthermore, our flow cytometric analysis employing Annexin V-IF647/PI double staining revealed that silencing SMYD5 significantly promoted apoptotic cell death in both early and late stages (Figure 3f). Specifically, the proportion of apoptotic cells increased from 24.1 ± 0.6% to 50.0 ± 7.4% in HepG2 cells and from 10.3 ± 0.8% to 19.5 ± 1.3% in Hep3B cells following the SMYD5 knockdown. These findings indicate a crucial role for SMYD5 in the modulation of apoptosis, suggesting that LIHC cells may exploit a high SMYD5 expression to evade programmed cell death. This ability to evade apoptosis is particularly pertinent in cancer, as it contributes to both tumorigenesis and therapeutic resistance.

In our comprehensive co-expression and functional analyses, we identified 10 co-expressed genes and 100 differentially expressed genes associated with SMYD5 in LIHC (Supplementary Figures S4a and S5). Notably, the negative correlation between SMYD5 and GHR suggests that targeting GHR could provide a novel strategy to hinder LIHC progression while simultaneously modulating SMYD5 activity (Supplementary Figure S4b). Our GO and KEGG analyses revealed that SMYD5 is involved in diverse biological processes, particularly those related to embryonic development and neuromodulation (Figure S4c). This discovery highlights potential pathways for therapeutic interventions aimed at modulating SMYD5’s influence on tumor biology. GSEA further suggests that lower SMYD5 expression correlates with the downregulation of metabolic pathways, including fatty acid metabolism and bile acid biosynthesis (Figure S4d), positioning SMYD5 as a crucial factor in maintaining metabolic homeostasis in LIHC. Importantly, our analysis reveals a significant positive correlation between higher SMYD5 levels and TILs, suggesting that SMYD5 may modulate the tumor immune microenvironment (Supplementary Figure S3). This is corroborated by violin plots indicating that higher SMYD5 levels correlate with elevated TIL scores (Supplementary Figure S3b). Given the immune system’s critical role in tumor regulation, targeting SMYD5 could enhance anti-tumor immunity and stand as a viable strategy in cancer immunotherapy.

Additionally, we have demonstrated for the first time that SMYD5 interacts with BRD4, a known enhancer of oncogene expression [26,27,28] (Figure 4). Co-immunoprecipitation experiments confirmed this interaction, suggesting a competitive relationship where BRD4 inhibition with (+)-JQ1 disrupts the SMYD5/BRD4 complex (Figure 4d). Consequently, combined targeting of SMYD5 and BRD4 may enhance anti-tumor effects, reinforcing the potential for dual-targeted approaches. Our results demonstrated that silencing SMYD5 significantly reduces the migratory and invasive capacities of Hep3B cells, suggesting that it may facilitate tumor dissemination (Figure 5). Notably, combining SMYD5 knockdown with BRD4 inhibition using (+)-JQ1 enhances anti-tumor effects significantly, indicating a synergistic interaction that could be exploited for therapeutic benefit. The significant rise in apoptosis following (+)-JQ1 treatment in SMYD5-silenced cells points to a critical interaction between these pathways. Flow cytometry analysis showed that silencing SMYD5 increases the apoptosis rates, suggesting that SMYD5 may contribute to the resistance to programmed cell death, a common feature of cancer progression. Previous studies have reported SMYD5-mediated K20 trimethylation of the ribosomal protein L40 [8]. More recent literature and our data suggest that SMYD5 also participates in histone methylation [9], potentially in concert with BRD4. The increased sensitivity of SMYD5-silenced Hep3B cells to BRD4 inhibition indicates that targeting SMYD5 can amplify the effectiveness of BRD4 inhibitors. The knockdown of SMYD5 significantly reduced its nuclear localization, as demonstrated by both immunofluorescence and nuclear fractionation Western blot assays (Figure 6a,b). Moreover, we observed that a SMYD5 knockdown affects the expression of key cell cycle regulators, notably CDK4 (Figure 6c,d). The decrease in the phosphorylated form of Cyclin D1 at Thr286 with combined treatment emphasizes crucial regulatory mechanisms of cell cycle activity, suggesting that the dual targeting of SMYD5 and BRD4 disrupts not only cell migration and proliferation but also induces apoptosis. The reduction in histone methylation marks H3K4me1, H3K4me2, and H3K4me3 upon targeting both SMYD5 and BRD4 is a novel finding (Figure 6). These alterations might lead to broader epigenetic reprogramming of tumor cells, which is crucial for regulating gene expression, and could decrease their malignant potential.

While our study presents significant insights, several limitations warrant careful consideration. Although our findings suggest a connection between SMYD5 and BRD4, we have not directly validated BRD4’s role through gene silencing or functional assays. Future studies should focus on direct validation methods, such as BRD4 knockdown, to clarify its precise role within the SMYD5 signaling pathway. Additionally, our investigations linking SMYD5 with immune cell types rely heavily on correlative analyses. Experimental validation in SMYD5-modulated cells will be essential to establish causal relationships. Furthermore, a major limitation of this study is the lack of in vivo validation, which would provide a more comprehensive understanding of SMYD5’s functional role in the tumor microenvironment. Further mechanistic investigations are necessary to elucidate the molecular pathways by which SMYD5 influences tumor behavior.

## 4. Materials and Methods

### 4.1. SMYD5 Pan-Cancer Analysis

Differential expression, paired comparison, OS, and DFS analyses of SMYD5 across various tumor types were performed using the TIMER2.0 (http://timer.cistrome.org, accessed on 17 April 2025) and GEPIA2 (http://gepia2.cancer-pku.cn, accessed on 17 April 2025) online platforms, with data sourced from TCGA [10,11,12]. Additionally, multivariate Cox regression analyses were conducted using R software (version 4.4.2) to evaluate the association between SMYD5 expression and patient prognosis across different cancer types.

### 4.2. SMYD5 Single-Gene Analysis in LIHC

RNA-Seq and corresponding clinical data for LIHC patients were obtained from the TCGA GDC database (https://portal.gdc.cancer.gov, accessed on 26 October 2024) [13]. SMYD5 expression data were extracted from these datasets using Perl (v5.30.0) for subsequent correlation analyses. Comprehensive multi-dimensional analyses were performed in R (v4.4.2), including a PFS analysis, ROC curve plotting, gene co-expression circos plots, construction of a nomogram, clinical forest plots, boxplots for clinical parameters, univariate and multivariate analyses, GO enrichment analysis, GSEA, and KEGG pathway enrichment analyses [29,30,31]. Additionally, immunohistochemical images of SMYD5 in both tumor and adjacent normal tissues from LIHC patients were obtained from HPA [32].

### 4.3. PPI Network Development

PPI networks for SMYD5 were constructed using the STRING database (25/2/2025) to identify 50 experimentally validated SMYD5-interacting proteins. Network visualization and figure refinement were performed using Cytoscape (3.10.3) software [33,34,35]. The interactions among these proteins were computationally ranked based on betweenness centrality (BC), with node color and size proportionally representing the magnitude of the BC values.

### 4.4. Cell Culture

HEK293T, Hep3B, and HepG2 cells were obtained from Procell (Austin, TX, USA) and cultured in Dulbecco’s Modified Eagle Medium (DMEM) supplemented with 10% fetal bovine serum (FBS). Human hepatic stellate cells LX-2 (Procell) were maintained in a specialized medium according to the manufacturer’s instructions. All cells were incubated at 37 °C in a humidified atmosphere containing 5% CO_2_ and 95% air. Cells were passaged every two days.

### 4.5. shRNAs and Plasmid Extraction

The shRNA sequence targeting SMYD5 (shSMYD5) was 5’-CTG TGA CAC TCT GGA GTT GAA-3′, and the negative control shRNA (shNC) sequence was 5′-UUC UCC GAA CGU GUC ACG UdTdT-3′. Plasmid amplification was performed using *E. coli* strain TOP10, and plasmids were purified with the EndoFree Mini Plasmid Kit II (TIANGEN, Beijing, China), according to the manufacturer’s protocol.

### 4.6. Cell Transfection to Establish SMYD5 Knockdown Cell Lines

Lentiviral particles containing shNC or shSMYD5 plasmids were generated in HEK293T cells using the packaging plasmids PMD2G and PsPAX2. Hep3B and HepG2 cells were infected with the viral supernatant diluted 1:1 (*v*/*v*) with DMEM basal medium. After 24 h, the medium was replaced with DMEM supplemented with 10% FBS. GFP fluorescence was monitored by microscopy three days post-infection to assess transduction efficiency. For selection of stable cell lines, the culture medium was replaced with DMEM containing 1 μg/mL puromycin and 10% FBS, and cells were subjected to selection for at least three days to establish Hep3B and HepG2 cell lines stably expressing shNC or shSMYD5.

### 4.7. Colony Formation Assay

A total of 1000 cells were seeded per well in 6-well plates and cultured until visible colonies formed. Cells were then fixed with paraformaldehyde for 30 min at room temperature, followed by staining with 0.1% crystal violet for 2 min. Excess stain was removed by rinsing the plates thoroughly with PBS. Plates were then inverted and air-dried prior to imaging for subsequent analysis.

### 4.8. Acquiring Protein Lysate

Protein lysates were prepared by combining RIPA buffer (Beyotime, Shanghai, China), protease inhibitor (Solarbio, Beijing, China), phosphatase inhibitor (MCE, Monmouth Junction, NJ, USA), and PMSF (Fdbio, Hangzhou, China) at a volumetric ratio of 100:1:1:1. Hep3B cells were lysed in this buffer on ice for 30 min. Cell suspensions were then sonicated for a total of 30 s (10 s per cycle), followed by centrifugation at 12,000 rpm for 10 min at 4 °C. All procedures were performed on ice to maintain protein integrity.

### 4.9. Transwell Assay

For the invasion assay, Matrigel (Yeason, Shanghai, China) was diluted 1:8 (*v*/*v*) with DMEM on ice. Sixty microliters of the diluted Matrigel were added to the upper chamber of the Nunc™ (Thermo Fisher Scientific, Waltham, MA, USA) polycarbonate cell culture inserts. After incubation at 37 °C for 3 h to allow matrix membrane formation, 100 μL of DMEM was added to each chamber for hydration. Subsequently, 5 × 10^4^ Hep3B sh-NC or sh-SMYD5 cells were seeded into each insert and cultured for 36 h. After incubation, the cells were fixed with paraformaldehyde for 30 min and stained with crystal violet for 5 minutes. Any non-invading cells on the upper side of the membrane were then removed with a cotton swab. The inserts were then photographed and analyzed using an inverted microscope.

### 4.10. MTT Assay

Hep3B sh-NC and sh-SMYD5 cells in the logarithmic growth phase were seeded into 96-well plates at a density of 6 × 10^3^ cells per well. Cells were treated with a series of (+)-JQ1 concentrations (0, 0.03, 0.1, 0.3, 1, 3, 10, and 30 μM) for 72 h. Cell viability was then assessed using an MTT assay (Solarbio) according to the manufacturer’s instructions to determine the sensitivity of Hep3B cell lines to (+)-JQ1.

### 4.11. Western Blot

Protein lysates were separated by 8% SDS-PAGE and transferred onto 0.22 μm PVDF membranes. Membranes were blocked with 5% BSA (Servicebio, Wuhan, China) for 1–2 h at room temperature. After blocking, membranes were appropriately trimmed and incubated overnight at 4 °C with primary antibodies in centrifuge tubes on a shaker. The following day, membranes were incubated with the appropriate secondary antibodies for 30 min to 1 h at room temperature. Detection was performed using an enhanced chemiluminescence (ECL) kit (Fdbio).

### 4.12. Co-IP Assay

Protein lysates from the IP and IgG control groups were incubated overnight at 4 °C with A/G magnetic beads (MCE) andthe SMYD5 antibody in 1.5 mL microcentrifuge tubes on a shaker. The magnetic beads were then washed four times with 400 μL PBST containing 0.5% Tween 20 using a magnetic rack. Bound proteins were eluted by denaturation to obtain samples for SDS-PAGE analysis.

### 4.13. Flow Cytometry

Apoptosis was assessed using the Annexin V-IF647/PI Cell Apoptosis Detection Kit (Servicebio) according to the manufacturer’s instructions. Hep3B cells were centrifuged at 500× *g* for 5 min and washed twice with pre-cooled PBS. The cell concentration was adjusted to approximately 1 × 10^5^ cells per tube with 1× Annexin V Binding Buffer. Cells were stained with 5 μL Annexin V-IF647 and 5 μL PI per 100 μL suspension for 8 min at room temperature in the dark. Fluorescence signals were detected on a BD Bioscience FACSCanto cytometer. Data were analyzed using FlowJo 10.10.0, and statistical analysis was performed with GraphPad Prism 9.5.1.

### 4.14. Immunofluorescence for SMYD5 Nucleus Translocation

To assess the expression and localization of SMYD5 in Hep3B cells, we performed immunofluorescence staining. Hep3B cells were fixed with 4% paraformaldehyde, permeabilized with 0.1% Triton X-100, and blocked with 5% BSA. Cells were incubated overnight with a primary antibody against SMYD5, followed by a fluorescently labeled secondary antibody. Nuclei were counterstained using DAPI. Images were captured using a Zeiss LSM 800 confocal microscope to visualize SMYD5 expression (green) and nuclear morphology (blue).

### 4.15. Nucleus Protein Extraction

Nuclear and cytoplasmic protein fractions were extracted using the Nuclear Protein Extraction Kit (Beyotime) following the manufacturer’s protocol. Briefly, Hep3B sh-NC and sh-SMYD5 cells were washed with PBS, collected by scraping or EDTA treatment, and pelleted by centrifugation. The cell pellet was incubated with a cytoplasmic extraction buffer containing PMSF, vortexed vigorously, and incubated on ice. After the addition of buffer B and further vertexing, samples were centrifuged at 15,000× *g* for 5 min at 4 °C to separate cytoplasmic proteins. The remaining pellet was washed, then resuspended in nuclear extraction buffer with PMSF, vortexed intermittently on ice for 30 min, and centrifuged at 15,000× *g* for 10 min at 4 °C to obtain nuclear proteins. Protein concentrations were determined, and samples were further analyzed by Western blot.

### 4.16. Image Processing

Quantification of the gray values from Western blot bands and colony numbers from the colony formation assay was performed using ImageJ software (version 2.1.0, NIH).

### 4.17. Statistical Analysis

All experimental data were statistically analyzed using either the t-test or one-way ANOVA in GraphPad Prism 9.5.1.

### 4.18. Antibodies in This Research

Different dilutions of the antibodies used in this article and merchant information can be found in Supplementary Table S1.

## 5. Conclusions

In summary, our research identifies SMYD5 as a significant oncogenic driver in LIHC, contributing to its progression through mechanisms that involve alterations in key epigenetic markers and interactions with BRD4. The high expression levels of SMYD5 not only correlate with adverse clinical outcomes but also play an active role in modulating the tumor microenvironment, influencing immune responses and tumor cell behavior. These findings advocate for further exploration into targeting the SMYD5-BRD4 interaction as a dual therapeutic approach in treating LIHC, with the potential to enhance treatment efficacy and overcome existing therapeutic resistances. Future studies should focus on in vivo models to validate these results and explore the translational perspectives of SMYD5 as a therapeutic target in LIHC.

## Data Availability

The original contributions of this study are available within the article and Supplementary Materials. For any further inquiries, please contact the corresponding author.

## Supplementary Materials

The following supporting information can be downloaded at: https://www.mdpi.com/article/10.3390/ph18081105/s1. Figure S1. Comparison of SMYD5 expression levels in paired clinical cancer samples. Figure S2. Clinical significance of SMYD5 expression in LIHC. (a) Nomogram incorporating SMYD5 for LIHC prognosis. (b) Calibration curves of the SMYD5-based nomogram. (c) ROC curve evaluating SMYD5 as a diagnostic marker in LIHC. (d) Univariate analysis of clinical variables and SMYD5 expression. (e) Multivariate analysis of clinical variables in LIHC. (f) Clinically relevant heatmap of SMYD5 in LIHC. (g) SMYD5 expression across different histological grades. (h) SMYD5 expression across clinical stages. (i) SMYD5 expression across T classification. Statistical significance: * *p* < 0.05, ** *p* < 0.01, *** *p* < 0.001. Figure S3. SMYD5 regulates immune infiltration in LIHC. (a) Tumor mutation burden in relation to SMYD5 expression in LIHC. (b) TME score stratified by SMYD5 expression in LIHC. (c) Correlation between SMYD5 expression and immune cell infiltration in LIHC. (d) Correlation between SMYD5 and immune checkpoint genes in LIHC tissues. Statistical significance: * *p* < 0.05, ** *p* < 0.01, *** *p* < 0.001. Figure S4. SMYD5 relates to oncogenic pathways in LIHC. (a) Co-expression network of SMYD5 and functionally related genes in LIHC. (b) KEGG pathway enrichment analysis of SMYD5-associated DEGs. (c) GO analysis of biological processes enriched in SMYD5-associated DEGs. (d) GSEA of SMYD5-associated transcriptional signatures in LIHC. Statistical significance: * *p* < 0.05, ** *p* < 0.01, *** *p* < 0.001. Figure S5. Differentially expressed genes correlated with SMYD5 in LIHC. Figure S6. SMYD5 PPI network analysis. Figure S7. Co-IP analysis demonstrates the interaction between SMYD5 and p53. Table S1. Different dilutions of the antibodies used in this article and merchant information.
