# Supplementary material for: SMYD5-BRD4 Interaction Drives Hepatocellular Carcinoma Progression: A Combined in Silico and Experimental Analysis
Source: Pharmaceuticals (Basel). 2025 Jul 25;18(8):1105. doi: 10.3390/ph18081105 (PMC12389522; doi:10.3390/ph18081105)
Supplement: Supplementary file 1 [file pharmaceuticals-18-01105-s001.zip › Supplementary Figures-0630 final.pdf]

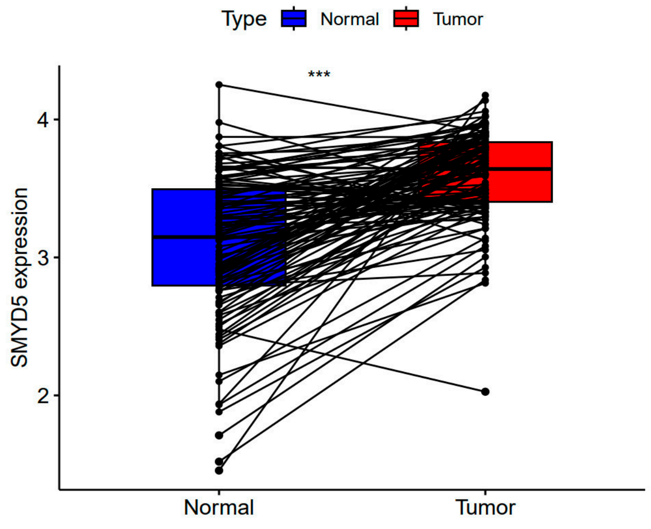

Supplementary Figure S1. Comparison of SMYD5 expression levels in paired clinical cancer samples.

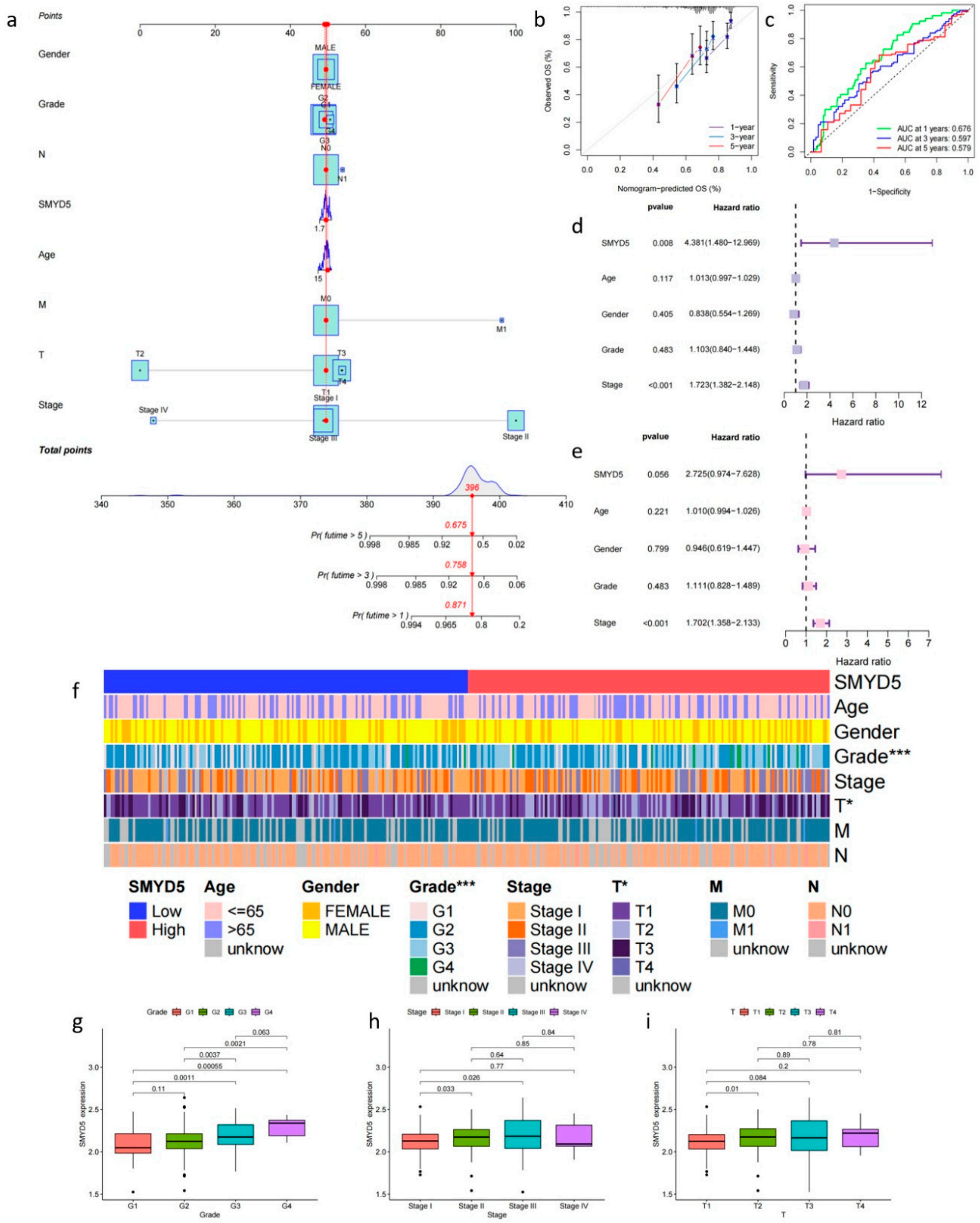

Supplementary Figure S2. Clinical significance of SMYD5 expression in LIHC. (a) Nomogram incorporating SMYD5 for LIHC prognosis. (b) Calibration curves of the SMYD5-based nomogram. (c) ROC curve evaluating SMYD5 as a diagnostic marker in LIHC. (d) Univariate analysis of clinical variables and SMYD5 expression. (e) Multivariate analysis of clinical variables in LIHC. (f) Clinically relevant heatmap of SMYD5 in LIHC. (g) SMYD5 expression across different histological grades. (h) SMYD5 expression across clinical stages. (i) SMYD5 expression across T classification. Statistical significance: *$p < 0.05$, **$p < 0.01$, ***$p < 0.001$.

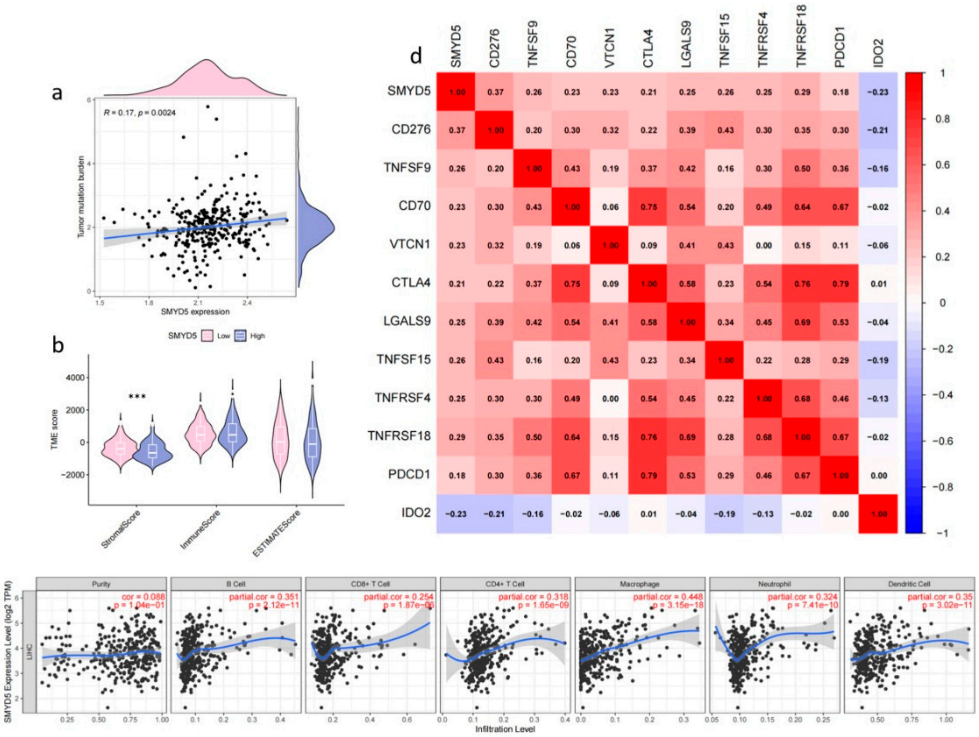

Supplementary Figure S3. SMYD5 regulates immune infiltration in LIHC. (a) Tumor mutation burden in relation to SMYD5 expression in LIHC. (b) TME score stratified by SMYD5 expression in LIHC. (c) Correlation between SMYD5 expression and immune cell infiltration in LIHC. (d) Correlation between SMYD5 and immune checkpoint genes in LIHC tissues. Statistical significance: *$P < 0.05$, **$P < 0.01$, ***$P < 0.001$.

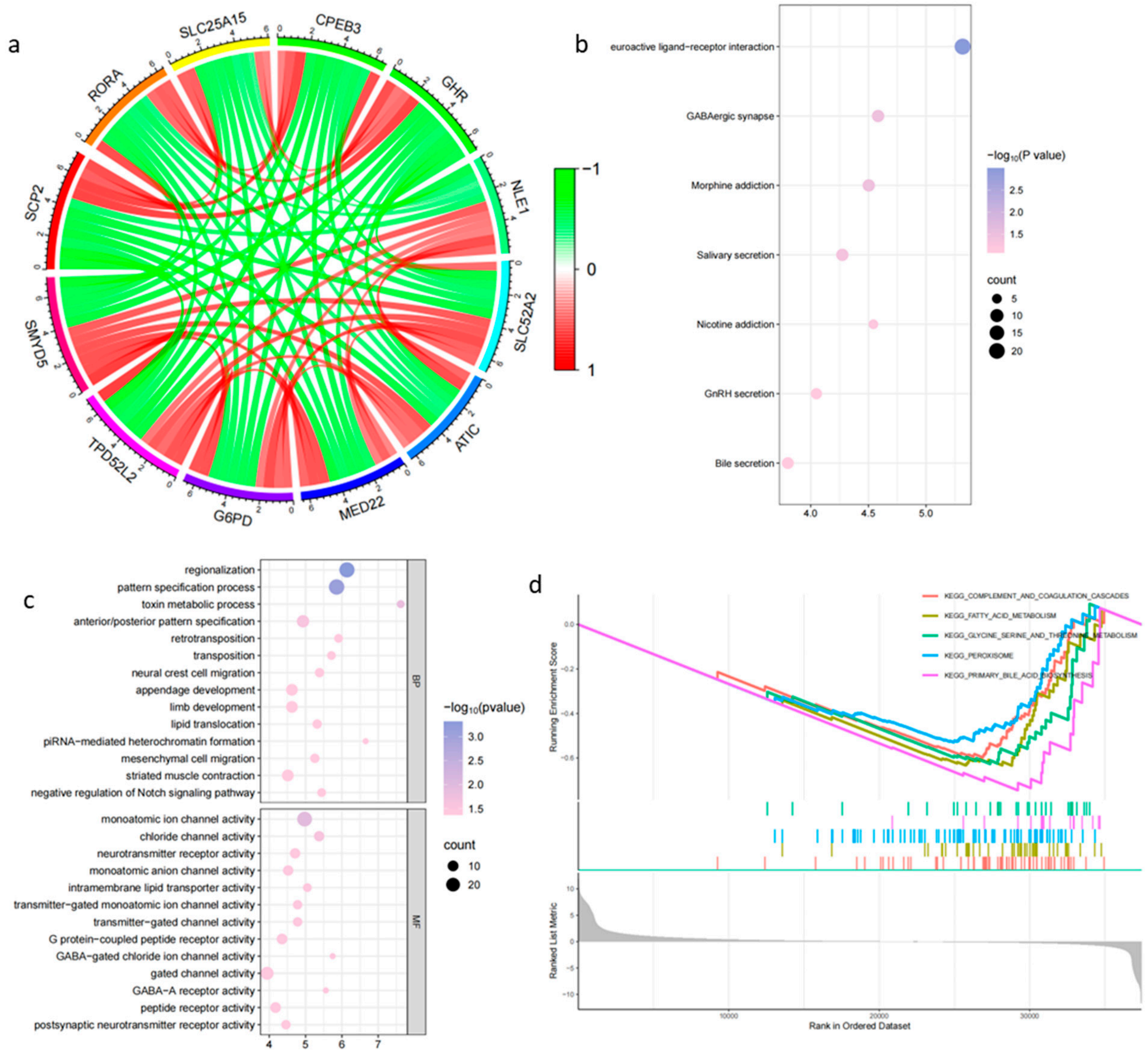

Supplementary Figure S4. SMYD5 relates to oncogenic pathways in LIHC. (a) Co-expression network of SMYD5 and functionally related genes in LIHC. (b) KEGG pathway enrichment analysis of SMYD5-associated DEGs. (c) GO analysis of biological processes enriched in SMYD5-associated DEGs. (d) GSEA of SMYD5-associated transcriptional signatures in LIHC. Statistical significance: *$p < 0.05$, **$p < 0.01$, ***$p < 0.001$.

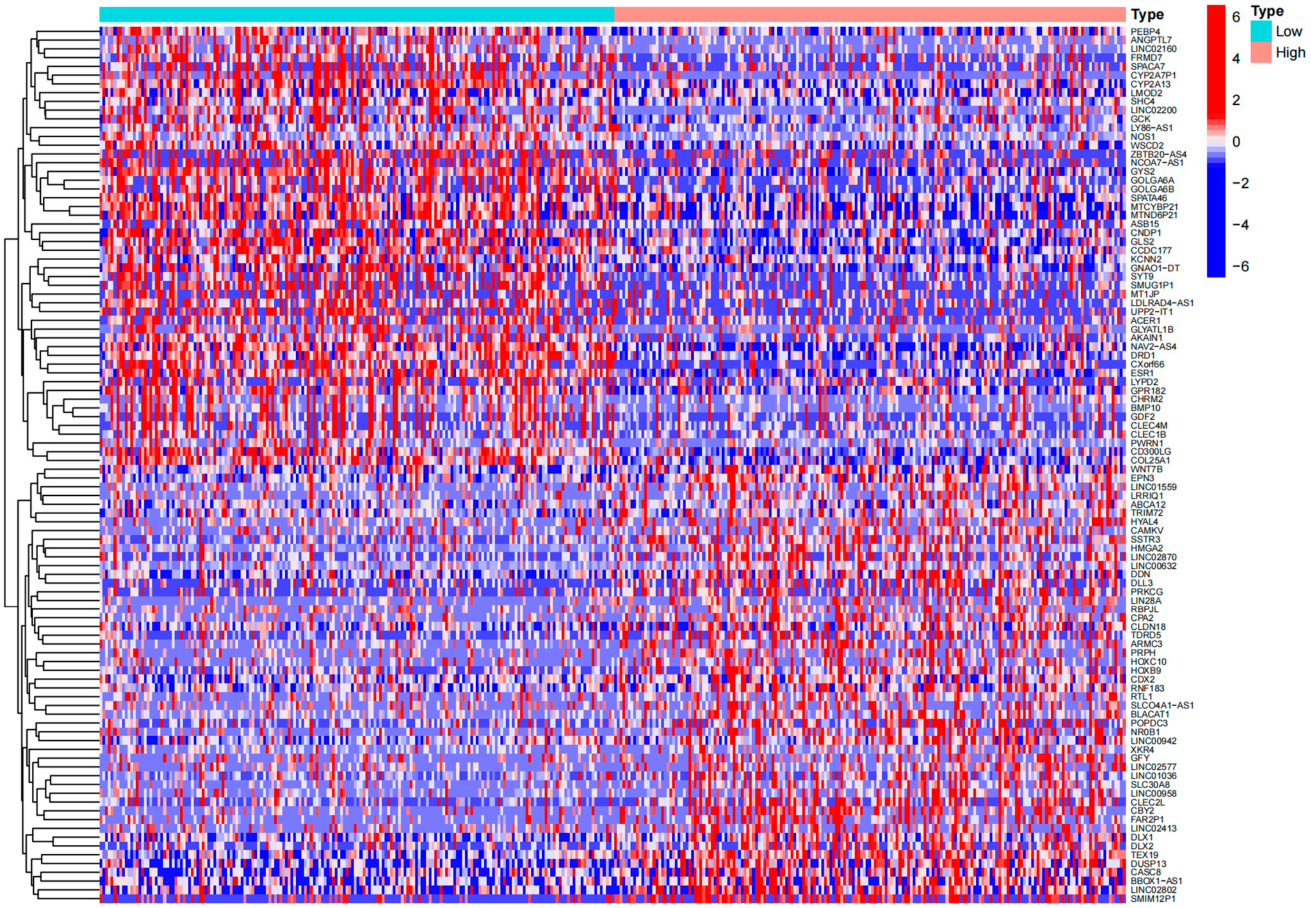

Supplementary Figure S5. Differentially expressed genes correlated with SMYD5 in LIHC.

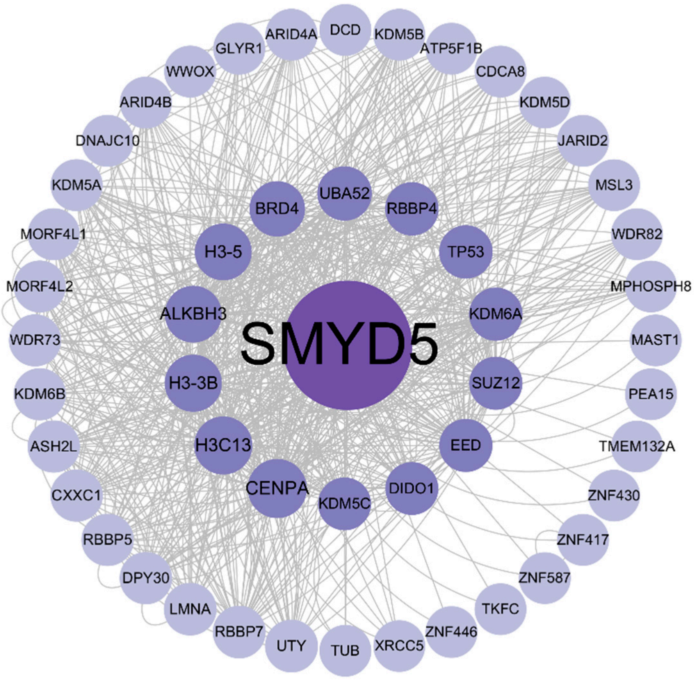

Supplementary Figure S6. SMYD5 PPI network analysis.

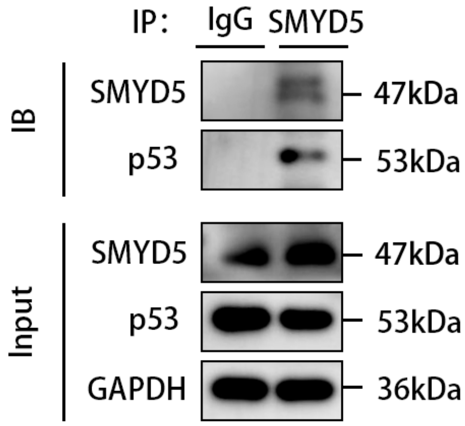

Supplementary Figure S7. Co-IP analysis demonstrates the interaction between SMYD5 and p53.
