# Supplementary material for: SMYD5-BRD4 Interaction Drives Hepatocellular Carcinoma Progression: A Combined in Silico and Experimental Analysis
Source: Pharmaceuticals (Basel). 2025 Jul 25;18(8):1105. doi: 10.3390/ph18081105 (PMC12389522; doi:10.3390/ph18081105)
Supplement: Supplementary file 1 [file pharmaceuticals-18-01105-s001.zip › Supplementary Table S1-Antibodies through this paper.pdf]

Supplementary Table S1. Different dilutions of the antibodies used in this article and merchant information

WB	Vendor	Dilution ratio
SMYD5	Abmart	1:800
BRD4	GeneTex	1:1500
GAPDH	Fdbio	1:5000
Tubulin	Fdbio	1:5000
	Co-IP	
IP		
SMYD5	Abmart	1:200
Anti-Rabbit IgG	Servicebio	1:800
IB		
SMYD5	Abmart	1:800
BRD4	Abmart	1:1000
GAPDH	Fdbio	1:5000
CDK4	SAB	1:1000
CyclinD1	SAB	1:1000
p-D1(Ser90)	SAB	1:1000
p-D1(Thr286)	SAB	1:1000
H3K4me1	SAB	1:1000
H3K4me2	SAB	1:1000
H3K4me3	SAB	1:1000
